# Astragaloside IV ameliorates acute pancreatitis in rats by inhibiting the activation of nuclear factor-κB

**DOI:** 10.3892/ijmm.2015.2070

**Published:** 2015-01-16

**Authors:** LEI QIU, GUOJIAN YIN, LI CHENG, YUTING FAN, WENQIN XIAO, GE YU, MIAO XING, RONGRONG JIA, RUIQING SUN, XIUYING MA, GUOYONG HU, XINGPENG WANG, MAOCHUN TANG, YAN ZHAO

**Affiliations:** 1 Department of Gastroenterology, Shanghai Tenth People’s Hospital, Tongji University School of Medicine, Shanghai 200072, P.R. China; 2 Department of Gastroenterology, Shanghai First People’s Hospital, Shanghai JiaoTong University, Shanghai 200080, P.R. China

**Keywords:** acute pancreatitis, astragaloside IV, inflammatory cytokine, nuclear factor-κB, superoxide dismutase

## Abstract

This study aimed to investigate the effects of astragaloside IV (AS-IV; 3-O-β-D-xylopyranosyl-6-O-β-D-glucopyranosylcycloastragenol), which has been reported to have comprehensive pharmacological functions, on sodium taurocholate (NaTc)/L-arginine (L-Arg)-induced acute pancreatitis (AP) in rats *in vivo* and in rat pancreatic acinar cells *in vitro*. NaTc-induced experimental AP was induced in rats by injecting 4% NaTc (0.1 ml/100 g) in the retrograde direction of the biliopancreatic duct. L-Arg-induced experimental AP was induced in rats by 2 intraperitoneal injections of 20% L-arg (3 g/kg), with an interval of 1 h between the injections. The rats were pre-treated AS-IV (50 mg/kg) or the vehicle (DMSO) 2 h prior to the induction of AP. Enzyme-linked immunosorbent assay, H&E staining, myeloperoxidase (MPO) activity, reverse transcription-quantitative PCR, western blot analysis and immunohistochemistry were used to evaluate the effects of AS-IV on AP. The results revealed that treatment with AS-IV significantly reduced serum amylase and lipase levels, pancreatic pathological alterations, the secretion of pro-inflammatory cytokines, MPO activity, and the protein expression of nuclear factor-κB (NF-κB) *in vivo*. Moreover, pre-treatment with AS-IV significantly increased the expression levels of manganese superoxide dismutase and cuprum/zinc superoxide dismutase. In the *in vitro* experiment, treatment of the cells with AS-IV aslo reduced rat pancreatic acinar cell necrosis and nuclear NF-κB activity, and enhanced the protein expression of superoxide dismutase. In conclusion, this study indicates that the protective effects of AS-IV on experimental AP in rats may be closely related to the inhibition of NF-κB. In addition, our results indicate that AS-IV may exert potential antioxidant effects on AP. Therefore, AS-IV may be an effective therapeutic agent for AP.

## Introduction

Acute pancreatitis (AP) is an inflammatory disease with high morbidity and mortality; however, the exact mechanisms involved are not yet fully understood. Although the majority of patients suffer mild or edematous AP with a low complication and mortality rate, 15–20% of patients develop severe AP (SAP) with a high mortality rate as high as 30% (1,2). The pathogenesis of AP is not clear; however, inflammatory cytokines, leukocytic infiltration, the activation of nuclear factor-κB (NF-κB) and oxidative stress are important factors (3,4). The inflammatory response is responsible for the morbidity and mortality associated with AP (5). During AP, particularly SAP, pancreatic acinar cells are the primary source of various pro-inflammatory cytokines, such as tumor necrosis factor-α (TNF-α), interleukin (IL)-1β and IL-6, which are directly responsible for aggravating the inflammatory response (5–9). These cytokines are upregulated from the initial phase of AP and directly correlate with many deleterious events locally in the pancreas and in distant organs (10,11). Among the multitude of inflammatory molecules, a key regulator of cytokine induction is the pleiotropic transcription factor, NF-κB (12). NF-κB is capable of regulating a variety of inflammatory mediators involved in AP, including TNF-α, IL-1β and IL-6. In most silent cells, NF-κB is kept inactive in the cytoplasm through sequestration in complexes with the inhibitor of NF-κB (IκB) proteins, such as inhibitory κBα (IκBα) and inhibitory κBβ (IκBβ) and it can be activated by the stimulation of nuclear translocation (9,13). The activation of NF-κB can promote the gene expression of TNF-α, IL-1β and IL-6, and plays a critical role in the initiation and perpetuation of AP (14). Movever, it has been suggested that oxidative stress plays a significant role in the pathogenesis of AP (15,16).

The saponin, astragaloside IV (AS-IV; a 3-O-β-D-xylopyranosyl-6-O-β-D-glucopyranosylcycloastragenol), which is purified from the known Chinese medical herb, *Astragalus membranaceus* (Fisch.) Bge. is one of the major and active components of *Αstragalus membranaceus*, which has been shown to have comprehensive pharmacological function (17,18). Diverse pharmacological activities have been found to be exerted by AS-IV such as anti-inflammatory, anti-oxidation, anti-infarction and antinociception (19–21). Previous studies have demonstrated the anti-inflammatory effects of AS-IV in a murine model of chronic asthma, in rats with focal cerebral ischemia/reperfusion injury, as well as *in vitro* (19,21,22). In addition, AS-IV has been shown to exert antioxidant effects through the reduction of free radicals, the inhibition of lipid peroxidation and the elevation of antioxidant enzymes (23).

To the best of our knowledge, to date, the protective effects of AS-IV on AP have not yet been investigated. Since the activation of NF-κB and oxidative stress are the major factors accounting for the pathogenesis of AP, we hypothesized that AS-IV may contribute to the prevention of AP progression. The aim of the present study was to investigate the protective effects of AS-IV in a rat model of AP. Our results revealed that AS-IV prevented the aggravation of AP by inhibiting the activition of NF-κB and counteracting oxidative stress, which suggests that AS-IV may be effective for the clinical therapy/prevention of AP.

## Materials and methods

### Ethics statement

All the animal-related procedures were approved by the Animal Care and Use Committee of the Shanghai Tenth People’s Hospital, Tongji University, Shanghai, China (permit no. 2011-RES1). This study was also approved by the Science and Technology Commission of Shanghai Municipality (ID: SYXK 2007-0006).

### Animals and materials

Male Sprague-Dawley rats weighing 250±30 g were purchased from the Shanghai SLAC Laboratory Animal Co., Ltd. (Shanghai, China). The animals were maintained under 12 h light-dark cycles at 22°C, provided with water *ad libitum*, fed standard laboratory chow and allowed to acclimatize for 1 week. The environment was maintained at a relative humidity of 30–70%. Purified AS-IV (>98%) was purchased from Shanghai Tauto Biotech Co., Ltd. (Shanghai, China). Sodium taurocholate (NaTc), L-arginine (L-Arg), dimethyl sulfoxide (DMSO) and hematoxylin and eosin (H&E) were purchased from Sigma-Aldrich (St. Louis, MO, USA). Antibodies against NF-κB p65, manganese superoxide dismutase (SOD1), cuprum/zinc superoxide dismutase (SOD2), β-actin and lamin-A were purchased from Abcam (Hong Kong, China). Peroxidase-conjugated secondary antibody was purchased from Santa Cruz Biotechnology, Inc. (Santa Cruz, CA, USA). Antibodies against IκBα and IκBβ were purchased from Cell Signaling Technology (CST; Shanghai, China).

### Experimental design

Before the experiment was initiated, the rats were fasted overnight with continued access to water. AS-IV, NaTc and L-Arg were dissolved in the vehicle (2% DMSO). NaTc-induced experimental AP was induced in the rats by injecting 4% NaTc (0.1 ml/100 g) in the retrograde direction of the biliopancreatic duct. The L-Arg-induced experimental AP was induced in the rats by 2 intraperitoneal injections of 20% L-Arg (3 mg/kg), with an interval of 1 h between the injections. In order to determine the optimal dose of AS-IV for the prevention of AP, we performed a preliminary experiment. A total of 36 rats were randomly divided into 9 groups (n=4 in each group) as follows: group 1, normal control; group 2, NaTc + vehicle-treated; groups 3–5, NaTc + AS-IV-treated (12.5, 25 and 50 mg/kg, respectively); group 6, L-Arg + vehicle-treated; and groups 7–9, L-Arg + AS-IV-treated (12.5, 25 and 50 mg/kg, respectively). The rats in the normal control group were injected with the vehicle intraperitoneally instead of 4% NaTc or 20% L-Arg. AS-IV, L-Arg and the vehicle (DMSO) were administered 2 h prior to the induction of AP. All the rats were sacrificed by taking blood from the heart 24 h after the induction of AP, a time point at which pancreatic damage had already been induced. The effects of AS-IV on AP were assessed by determining the serum amylase level and pancreatic staining with H&E, to obtain an optimal dose. Accordingly, the optimal dose of AS-IV (50 mg/kg) was used for the next series of experiments.

Subsequently, 90 rats were randomly divided into 5 groups (n=18 in each group) as follows: group 1, normal control; group 2, NaTc + vehicle-treated; group 3, NaTc + AS-IV-treated; group 4, L-Arg + vehicle-treated; and group 5, L-Arg + AS-IV-treated. The induction of AP and the administration of AS-IV or the vehicle were carried out in a similar manner as in the preliminary experiment. The rats were sacrificed by taking blood from the heart at 12, 24 and 48 h after the induction of AP, 6 rats at each time point in each group. Blood samples were obtained to determine the serum amylase, lipase and cytokine levels. A portion of the pancreas was rapidly removed from each rat and fixed in 4% neutral paraformaldehyde solution for morphological examination. The remaining portion of each pancreas was stored in liquid nitrogen for further analysis.

### Isolation of pancreatic acinar cells from rats

Pancreatic acinar cells were isolated from the rats using a collagenase digestion procedure as previously described (24). The isolated acinar cells were incubated at 37°C under humidified conditions of 95% air and 5% CO_2_ in Dulbecco’s modified Eagle’s medium/Ham’s F12 Medium (DMEM/F12) containing 10% fetal bovine serum (FBS) and 1% penicillin-streptomycin (all from Gibco-BRL, Grand Island, NY, USA) with or without NaTc (3,750 nmol/l)/L-Arg (40 *μ*mol/l) and AS-IV at different doses (20, 40, 80 and 160 *μ*mol/l). At 12 h following treatment with NaTc/L-Arg, the pancreatic acinar cells were used to carry out a series of experiments, including Cell Titer-Glo luminescent cell viability assa, cell counting kit-8 (CCK-8) assay and western blot analysis.

### Histological examination

The pancreatic tissue samples were fixed in 4% neutral paraformaldehyde solution for 24 h, dehydrated through a graduated ethanol series, embedded in paraffin blocks and cut into 5-*μ*m-thick sections. The pancreatic sections were dewaxed in xylene, hydrated through an upgraded ethanol series and stained with H&E. Morphological changes were observed under a light microscope (DMI6000B; Leica, Wetzlar, Germany). Ten microscopic fields were randomly selected to be observed in each paraffin section.

### Serum amylase, lipase and pro-inflammatory cytokine assay

Blood samples of each rat were maintained at 4°C for 24 h prior to centrifugation at 3,000 × g for 15 min at 4°C, and serum-stored at −80°C. The serum activities of amylase and lipase were measured by enzyme dynamics chemistry using commercial kits according to the manufacturer’s instructions in a Roche/Hitachi modular analytics system (Roche, Mannheim, Germany). Serum TNF-α, IL-1β and IL-6 levels were measured by enzyme-linked immunosorbent assay (ELISA) using a commercial kit (Quantikine; R&D Systems, Minneapoils, MN, USA).

### Meaurement of myeloperoxidase (MPO) activity

Neutrophil sequestration in the pancreas was quantified by measuring tissue MPO activity according to a previously described method (25). Pancreatic tissue samples were homogenized in 20 mM phosphate buffer (pH 7.4) and centrifuged (12,000 × g for 10 min at 4°C). The pellet was resuspended in 50 mM phosphate buffer (pH 6), containing 0.5% hexade cyltrimethylammonium bromide (HETAB). The suspension was subjected to 4 cycles of freezing and thawing and was further disrupted by sonication for 1 min. The sample was then centrifuged (12,000 × g for 5 min at 4°C). Aliquots of supernatant were added to the reaction mixture containing 0.167 mg/ml of o-dianisdine dihydrochloride and 0.0005% H_2_O_2_ solution, which were prepared in 50 mM of phosphate buffer. The change in absorbance at 450 nm was then measured for 5 min using a Beckman spectrophotometer (DU640B; Beckman Coulter, Brea, CA, USA). One unit of MPO activity was defined as that degrading 1 mmol of peroxide/min at 25°C. The activity was expressed as unit/milligram of tissue.

### Reverse transcription-quantitative (real-time) polymerase chain reaction (RT-qPCR)

The mRNA transcripts were analyzed by RT-qPCR of the pancreatic tissue. Total RNA was isolated using TRIzol reagent (Invitrogen, Carlsbad, CA, USA) following the manufacturer’s instructions and was subjected to reverse transcription using the Prime-Script RT reagent kit (Takara, Shiga, Japan). SYBR-Green quantitative PCR was performed using a 7900HT Fast Real-time PCR system (Applied Biosystems, Foster City, CA, USA) according to the instructions provided with SYBR Premix EX Taq (Takara). The relative mRNA levels were normalized to the mRNA levels of glyceraldehyde 3-phosphate dehydrogenase (GAPDH) and the fold change of each mRNA was calculated using the comparative CT (2^−ΔΔCT^) method. Primer sequences for these biomarkers were as follows: rat TNF-α forward, 5′-TGATCCGAGATGTGGAACTG-3′ and reverse, 5′-GGCCATGGAACTGATGAGAG-3′; rat IL-1β forward, 5′-CATCCAGCTTCAAATCTCAC-3′ and reverse, 5′-ACCACTTGTTGGCTTATGTT-3′; rat IL-6 forward, 5′-TCTCTCCGCAAGAGACTTCC-3′ and reverse, 5′-TCTTGGTCCTTAGCCACTCC-3′; rat IκBα forward, 5′-CATGAAGAGAAGACACTGACCA TGGAA-3′ and reverse, 5′-TGGATAGAGGCTAAGTGTAGACACG-3′; rat IκBβ forward, 5′-GCAGGAAGATGTGGTGGA-3′ and reverse, 5′-CTGGCTCATATGGTTTCC-3′; rat SOD1 forward, 5′-CACAAGCACAGCCTCCCTGA-3′ and reverse, 5′-GCAATCTGTAAGCGACCTTG-3′; and rat SOD2 forward, 5′-TTCGAGCAGAAGGCAAGCGG-3′ and reverse, 5′-GTACGGCCAATGATGGAATG-3′.

### Western blot analysis

For western blot analysis, the rat pancreas was rapidly ground in liquid nitrogen, as previously described (26). The resulting powder or isolated acinar cells were lysed in nuclear extract following the manufacturer’s instructions for the preparation of nuclear and cytoplasmic proteins (Pierce, Rockford, IL, USA). On the other hand, the resulting powder or isolated acinar cells were reconstituted in ice-cold RIPA buffer containing 1 mmol/l phenylmethanesulfonyl fluoride (PMSF, 1 mM) and a cocktail of protease inhibitors (1:100 dilution; Sigma-Aldrich). The samples were centrifuged at 4°C for 15 min at 10,000 × g and supernatants were recovered. The concentrations of nuclear, cytoplasmic and total proteins were determined using the BCA method (Pierce). A 50 *μ*g portion of protein or equal proportion of concentrated supernatant was subjected to sodium dodecyl sulfate/polyacrylamide gel electrophoresis (SDS-PAGE; Bio-Rad, Hercules, CA, USA), and then blotted following standard methods. Non-specific binding to the membrane was blocked by 5% (w/v) dry non-fat milk in Tris-buffered saline/0.05% Tween-20 (TBST) at room temperature for 1 h in a covered container. The blots were incubated overnight at 4°C with rabbit polyclonal anti-NF-κB p65 antibody (1:400 dilution; Abcam), rabbit polyclonal anti-IκBα antibody (1:200 dilution; CST), rabbit polyclonal anti-IκB β antibody (1:200 dilution; CST), rabbit polyclonal anti-SOD1 antibody (1:500 dilution; Abcam), rabbit polyclonal anti-SOD2 antibody (1:2,000 dilution; Abcam), rabbit polyclonal anti-lamin A antibody (1:500 dilution; Abcam) and mouse monoclonal anti-β-actin (1:1,000 dilution; Abcam) diluted in 5% BSA. Lamin A and β-actin were used as the internal reference for nuclear proteins and cytoplasmic proteins, respectively. The membranes were washed with TBST and incubated with a secondary rabbit anti-mouse IhG horseradish peroxidase (HRP) antibody (1:2,000; Santa Cruz Biotechnology, Inc.) or mouse anti-rabbit IgG-HRP antibody (1:2,000 dilution; Santa Cruz Biotechnology, Inc.) diluted in 5% (w/v) dry non-fat milk in TBST for 1 h at room temperature. Finally, the membranes were washed with TBST, developed using the ECL detection system (Santa Cruz Biotechnology, Inc.), quickly dried and exposed to ECL film.

### Immunohistochemistry

Formalin-fixed, paraffin-embedded samples were cut at a thickness of 5 *μ*m. Each tissue section was deparaffinized and rehydrated with graded ethanol. For antigen retrieval, the slides were boiled in EDTA (1 mM, pH 8.0) for 15 min in a microwave oven. Endogenous peroxidase activity was blocked with a 0.3% hydrogen peroxide solution for 10 min at room temperature. After rinsing with phosphate-buffered saline (PBS), the slides were incubated overnight at 4°C with the antibody against NF-κB p65 (1:100 dilution), washed with 3 times with 0.02% Tween-20 in PBS for 10 min each time and incubated with biotinylated secondary antibody diluted at 1:500 for 40 min. The antibody binding were detected with an Envision Detection kit, peroxidase/DAB, rabbit/mouse (GeneTech, Ltd., Shanghai, China). The sections were counterstained with hematoxylin. In general, NF-κB p65 was stained in the cytoplasm, and the translocation from the cytoplasm to the nucleus indicated the activation of NF-κB p65. The sections were observed under a light microscope (DMI6000B; Leica) at a magnification of x400.

### Quantification of cell viability

Pancreatic acinar cell necrosis was determined by measuring the adenosine triphosphate (ATP) levels using the Cell Titer-Glo Luminescent Cell Viability Assay kit (Promega, Madison, WI, USA) as previously described (27). Cell proliferation was determined by CCK-8 assay using a CCK-8 kit (Dojindo, Kumamoto, Japan), according to manufacturer’s instructions. The viability of the isolated rat pancreatic acinar cells was analyzed with the above 2 methods.

### Statistical analysis

All data are presented as the means ± SD. Statistical analysis was performed using the Student’s t-test for comparisons of 2 groups, and one way ANOVA was used to analyze the differences among multiple groups. A value of P<0.05 was considered to indicate a statistically significant difference. All analyses were conducted using statistical analysis software (SPSS 17.0).

## Results

### Prelilminary analysis

**Figure 1:**
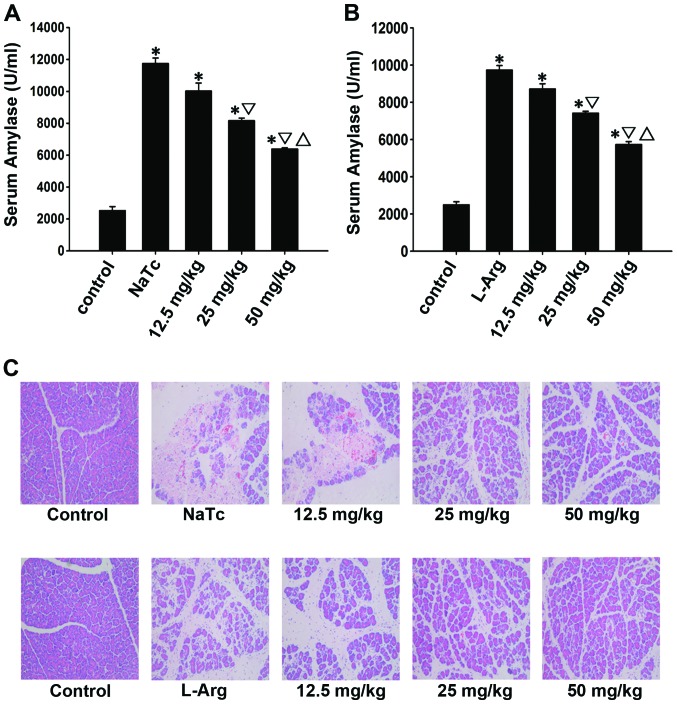
Results of the prelilminary experiment. The activity of AS-IV on AP was evaluated based on (A and B) the serum amylase level and (C) pancreatic histological alterations. In rats with NaTc/L-Arg-induced AP, the serum amylase levels were significantly increased compared with those of the normal control group (P<0.05). However, pre-treatment with the high dose (50 mg/kg) of AS-IV led to a more effective decrease in the serum amylase level compared with moderate dose (25 mg/kg) and low dose (12.5 mg/kg). Morever, the high dose (50 mg/kg) of AS-IV markedly reduced interstitial edema, inflammatory cell infiltration and acinar cell necrosis. Data are represented as the means ± SD from 3 independent experiments. ^*^P<0.05 compared with the normal control group at the same time point; ^▽^P<0.05 compared with the NaTc/L-Arg + vehicle-treated group at the same time point; ^△^P<0.05 compared with the 25 mg/kg NaTc/L-Arg + AS-IV-treated group. AS-IV, astragaloside IV; NaTc, sodium taurocholate; L-Arg, L-arginine; AP, acute pancreatitis.

The optimal effective dose of AS-IV was evaluated based on the serum amylase level and pancreatic H&E staining. In the prelilminary experiment (Fig. 1), the highest dose (50 mg/kg) used reduced pancreatic damage more prominently compared with the moderate dose (25 mg/kg) and the low dose (12.5 mg/kg) used at the time point of 24 h following the induction of AP (P<0.05). Therefore, the dose of 50 mg/kg was selected as the optimal dose for the following experiments.

### AS-IV alleviates the histopathological alterations of the pancreas

**Figure 2:**
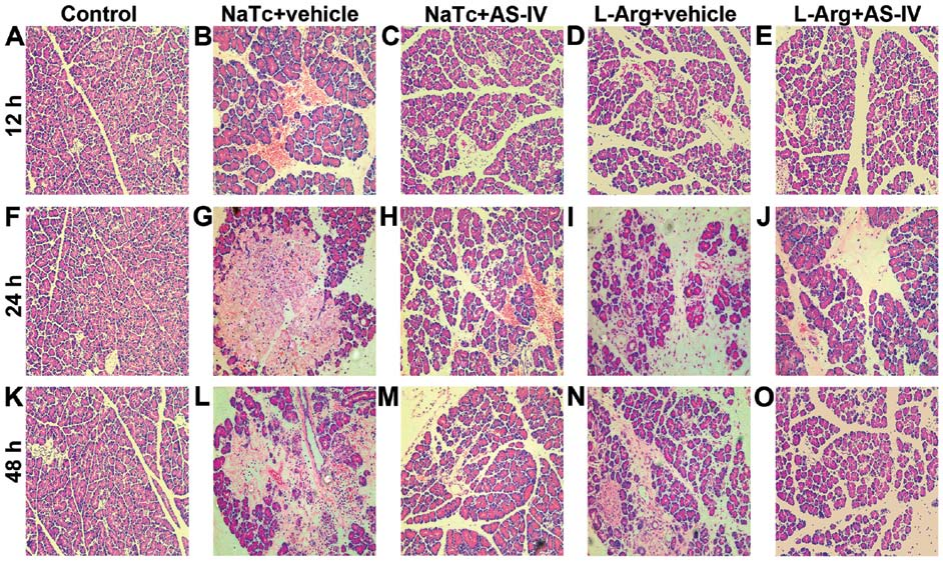
Pancreatic histological alterations. Rats were sacrificed at 12, 24 and 48 h after the induction of AP, 6 rats at each time point in each group. Representative light micrographs from (A, F and K) the normal control group, (B, G and L) NaTc + vehicle-treated group, (C, H and M) NaTc + AS-IV-treated group, (D, I and N) L-Arg + vehicle-treated group, and (E, J and O) L-Arg + AS-IV-treated group are presented (magnification, x200). By comparison, pre-treatment with AS-IV significantly decreased interstitial edema, inflammatory cell infiltration and acinar cell necrosis. AS-IV, astragaloside IV; NaTc, sodium taurocholate; L-Arg, L-arginine; AP, acute pancreatitis.

To determine the effects of AS-IV on the development and severity of AP, the rats were pre-treated with AS-IV (50 mg/kg) or the vehicle (DMSO) prior to the induction of AP. In the normal control group, the pancreatic histological characteristics were typical of a nomal architecture (Fig. 2A, F and K). A histological examination of the rat pancreas revealed that the rats in the NaTc + vehicle-treated group (Fig. 2B, G and L) and L-Arg + vehicle-treated group (Fig. 2D, I and N) showed massive edema, hemorrhaging, inflammatory cell infiltration and necrosis. Pre-treatment with AS-IV resulted in lower interstitial edema, less inflammatory cell infiltration and alleviatived acinar cell necrosis at 3 time points in the NaTc + AS-IV-treated group (Fig. 2C, H and M) and L-Arg + AS-IV-treated group (Fig. 2E, J and O). Therefore, pre-treatment with AS-IV observably reduced both the NaTc- and L-Arg-induced histological characteristics of pancreatic injury.

### AS-IV reduces serum amylase and lipase levels

**Figure 3:**
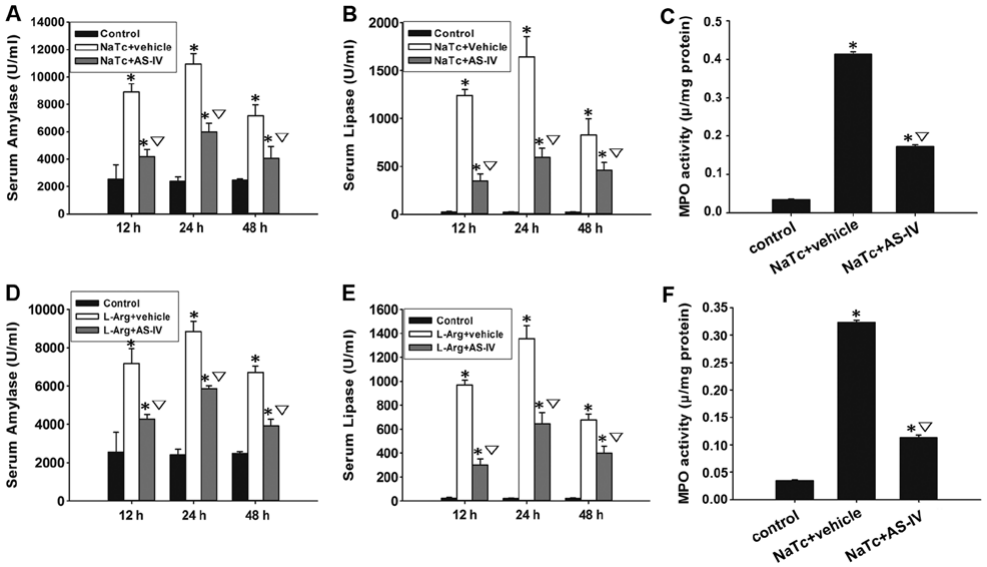
The levels of serum amylase, lipase and MPO activity. Rats were sacrificed at 12, 24 and 48 h after the induction of AP, 6 rats at each time point in each group. Serum was collected, and the levels of (A and D) serum amylase and (B and E) serum lipase were measured. AS-IV significantly reduced the levels of amylase and lipase in serum. (C and F) Rat pancreatic MPO activity was measured at the time point of 24 h after the induction of AP. AS-IV also reduced the NaTc/L-Arg-induced activity of MPO. Data are represented as the means ± SD from 3 independent experiments. ^*^P<0.05 compared with the normal control group at the same time point; ^▽^P<0.05 compared with the NaTc/L-Arg + vehicle-treated group at the same time point. MPO, myeloperoxidase; AS-IV, astragaloside IV; NaTc, sodium taurocholate; L-Arg, L-arginine; AP, acute pancreatitis.

Serum amylase and lipase are most commonly regarded as biochemical indicators of AP (28). Thus, we assessed the development of AP by measuring the serum amylase (Fig. 3A and D) and lipase levels (Fig. 3B and E). Compared to the normal control group, the serum amylase and lipase levels of the other 4 groups were increased significantly (P<0.05). In comparison to the NaTc/L-Arg + vehicle-treated groups, pre-treatment with AS-IV significantly reduced the elevation of the serum amylase and lipase levels at 3 time points (P<0.05).

### AS-IV decreases the secretion of pro-inflammatory cytokines and MPO activity

**Figure 4:**
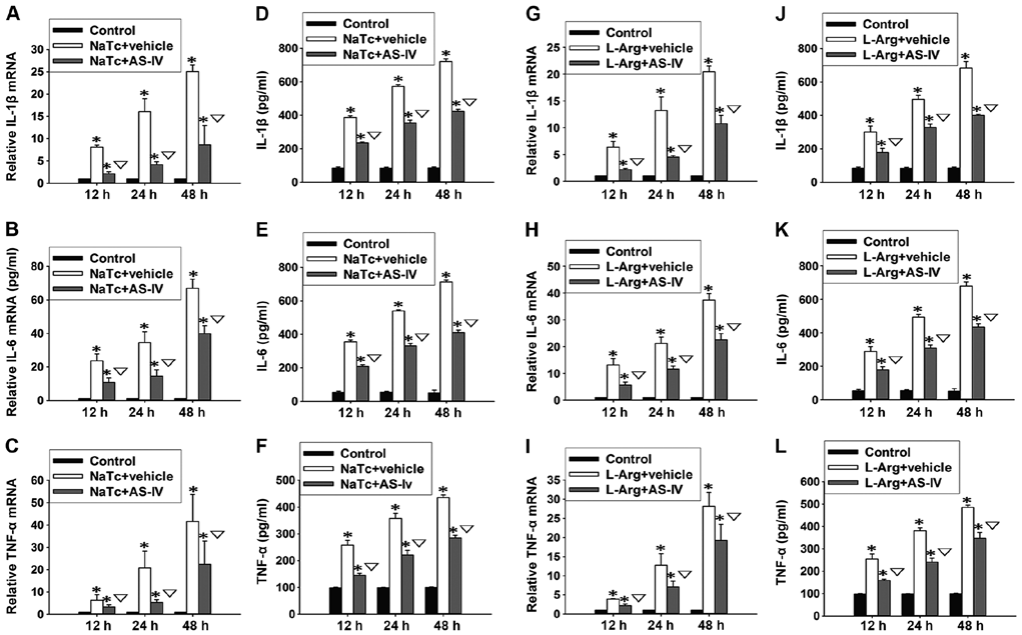
Secretion of pro-inflammatory cytokines. The serum levels of IL-1β, IL-6 and TNF-α were measured by ELISA at 3 time points for each group (D-F and J-L). The mRNA expression levels of IL-1β, IL-6 and TNF-α were detected by RT-qPCR at 3 time -points for each group (A-C and G-I). The production of these pro-inflammatory cytokines in serum and their pancreatic mRNA expression levels were decreased by AS-IV. Data are represented as the means ± SD from 3 independent experiments. ^*^P<0.05 compared with the normal control group at the same time point; ^▽^P<0.05 compared with the NaTc/L-Arg + vehicle-treated group at the same time point. AS-IV, astragaloside IV; NaTc, sodium taurocholate; L-Arg, L-arginine; IL-1β, interleukin-1β; IL-6, interleukin-6; TNF-α, tumor necrosis factor-α.

Neutrophil sequestration in the pancreas was determined by measuring MPO activity in the pancreatic tissue at the time point of 24 h after the induction of AP. Pre-treatment with AS-IV reduced the NaTc- or L-Arg-induced activity of MPO in the pancreatic tissue (Fig. 3C and F). Based on the results of ELISA and RT-qPCR, we found that both the mRNA expression levels (Fig. 4A-C and G-I) and the serum levels (Fig. 4D-F and J-L) of IL-1β, IL-6 and TNF-α in the normal control group were significantly lower than those of the other 4 groups at 3 time points (P<0.05). Compared with the NaTc + vehicle-treated group and the L-Arg + vehicle-treated group, pre-treatment with AS-IV significantly reduced the elevation of the serum levels and mRNA expression levels of IL-1β, IL-6 and TNF-α (P<0.05).

### AS-IV inhibits the nuclear translocation of NF-κB p65

**Figure 5:**
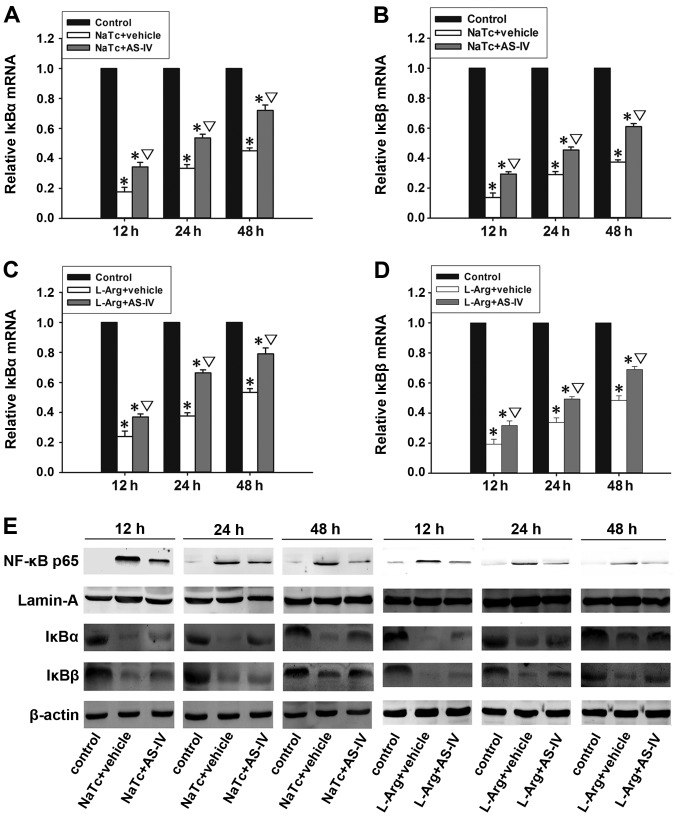
Western blot analysis of NF-κB expression. (A-D) The mRNA expression levels of IκBα and IκBβ were detected by RT-qPCR at 3 time points for each group. (E) The mRNA expression levels of IκBα and IκBβ in pancreatic tissue were decreased by AS-IV. Western blot analysis of NF-κB p65 in the nucleus, and IκBα, IκBβ in the whole protein at 3 time points for each group. Lamin A and β-actin were regarded as the internal reference for nuclear proteins and whole proteins, respectively. The administration of AS-IV increased the mRNA and protein expression levels of IκBα and IκBβ, and inhibited the activation of NF-κB. Figures are representative of 3 independent experiments. Data are represented as the means ± SD from 3 independent experiments. ^*^P<0.05 compared with the normal control group at the same time point; ^▽^P<0.05 compared with the NaTc/L-Arg + vehicle-treated group at the same time point. AS-IV, astragaloside IV; NaTc, sodium taurocholate; L-Arg, L-arginine; NF-κB, nuclear factor-κB.

**Figure 6:**
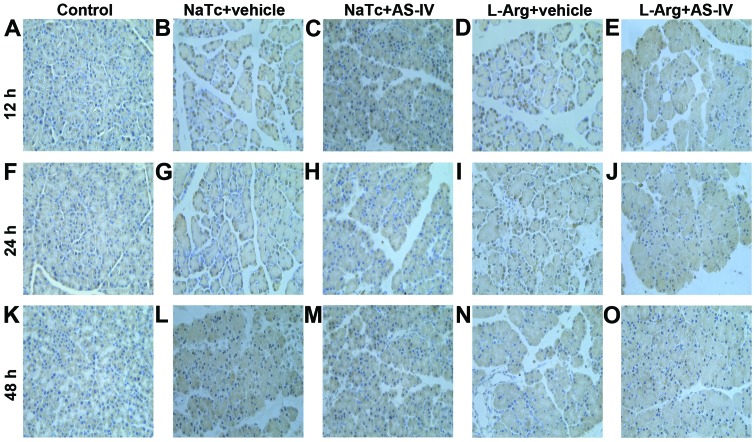
Immunohistochemical analysis of NF-κB expression. (A, F and K) Normal control group, (B, G and L) NaTc + vehicle-treated group, (C, H and M) NaTc + AS-IV-treated group, (D, I and N) L-Arg + vehicle-treated group, and (E, J and O) L-Arg + AS-IV-treated group (original magnification, x400). Pre-treatment with AS-IV significantly decreased the staining intensity of NF-κB in the nucleus, particularly at the time point of 12 h after the induction of AP. Results are representative of 3 independent experiments. AS-IV, astragaloside IV; NaTc, sodium taurocholate; L-Arg, L-arginine; AP, acute pancreatitis; NF-κB, nuclear factor-κB.

The activation of NF-κB plays an important role in the induction of pro-inflammatory cytokines (12). Generally, inactivated NF-κB is sequestered by the inhibitor IκB in the cytoplasm, and it can be activated by stimulation with nuclear translocation (13). The mRNA expression levels of IκBα (Fig. 5A and C) and IκBβ (Fig. 5B and D) in the normal control group were significantly higher than those of the other 4 groups at 3 time points (P<0.05). In the NaTc + AS-IV-treated group and L-Arg + AS-IV-treated group, the mRNA expression levels of IκBα and IκBβ were significantly higher than those in the NaTc + vehicle-treated group and L-Arg + vehicle-treated group (P<0.05). In order to ascertain whether AS-IV inhibits the activation of NF-κB, we examined the protein expression levels of NF-κB p65 in the nucleus and the levels of IκBα and IκBβ in the cytoplasm by western blot analysis at 3 time points in each group (Fig. 5E). In the NaTc + vehicle-treated group and L-Arg + vehicle-treated group, the protein expression levels of IκBα and IκBβ were markedly reduced; however, the level of nuclear NF-κB p65 was significantly increased at the 3 time points, particularly at the time point of 12 h following the induction of AP. Compared with NaTc/L-Arg + vehicle-treated group at the 3 time points, the administration of AS-IV markedly decreased the degradation of IκBα and IκBβ, as well as the nuclear protein expression level of NF-κB p65. Subsequently, we further measured the level of NF-κB p65 in the nucleus by immunohistochemistry (Fig. 6). In the normal control group (Fig. 6A, F and K), immunoreactivity for NF-κB p65 protein was hardly observed in the nucleus. However, the NaTc + vehicle-treated group (Fig. 6B, G and L) and L-Arg + vehicle-treated group (Fig. 6D, I and N) exhibited a strong positive expression of NF-κB p65 in the nucleus, particularly at the time point of 12 h following the induction of AP. As expected, in the NaTc + AS-IV-treated group (Fig. 6C, H and M) and L-Arg + AS-IV-treated group (Fig. 6E, J and O), the positive expression of NF-κB p65 in the nucleus was weaker than that in the NaTc/L-Arg + vehicle-treated groups at the 3 time points. The results from western blot analysis and immunohistochemistry confirmed that AS-IV inhibited the nuclear translocation of NF-κB p65.

### Antioxidant effects of AS-IV on AP

**Figure 7:**
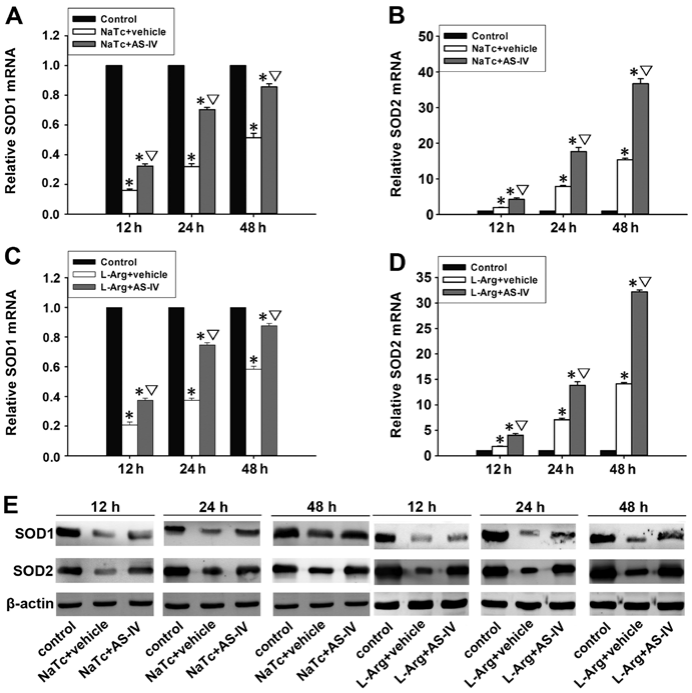
Antioxidant effects of AS-IV on AP. The mRNA expression levels of SOD1 and SOD2 were detected by RT-qPCR at 3 time points for each group (A-D). Data are represented as the means ± SD from 3 independent experiments. The protein expression levels of SOD1 and SOD2 in the whole protein were measured by western blot analysis at 3 time points for each group (E). β-actin was regarded as the internal reference for whole proteins. AS-IV significantly increased the mRNA and protein expression levels of SOD1 and SOD2. Figures are representative of 3 independent experiments. ^*^P<0.05 compared with the normal control group at the same time point; ^▽^P<0.05 compared with the NaTc/L-Arg + vehicle-treated group at the same time point. AS-IV, astragaloside IV; SOD1, manganese superoxide dismutase; SOD2, cuprum/zinc superoxide dismutase; NaTc, sodium taurocholate; L-Arg, L-arginine; AP, acute pancreatitis.

Oxidative stress imposed by reactive oxygen species (ROS) is considered one of the forerunners of AP (29). The antioxidant enzyme, SOD, plays an improtant role in protecting cells against the generation of ROS (30). The mRNA expression levels of SOD1 (Fig. 7A and C) and SOD2 (Fig. 7B and D) were detected by RT-qPCR. In the NaTc + AS-IV-treated group and L-Arg + AS-IV-treated group, the mRNA expression levels of SOD1 and SOD2 were significantly increased in comparison with the NaTc/L-Arg + vehicle-treated group (P<0.05). Subsequently, the protein expression levels of SOD1 and SOD2 in the pancreatic tissue were quantified by western blot analysis (Fig. 7E). As expected, pre-treatment with AS-IV significantly increased the protein expression levels of SOD1 and SOD2 in the pancreatic tissue.

### Effects of AS-IV on AP in vitro

**Figure 8:**
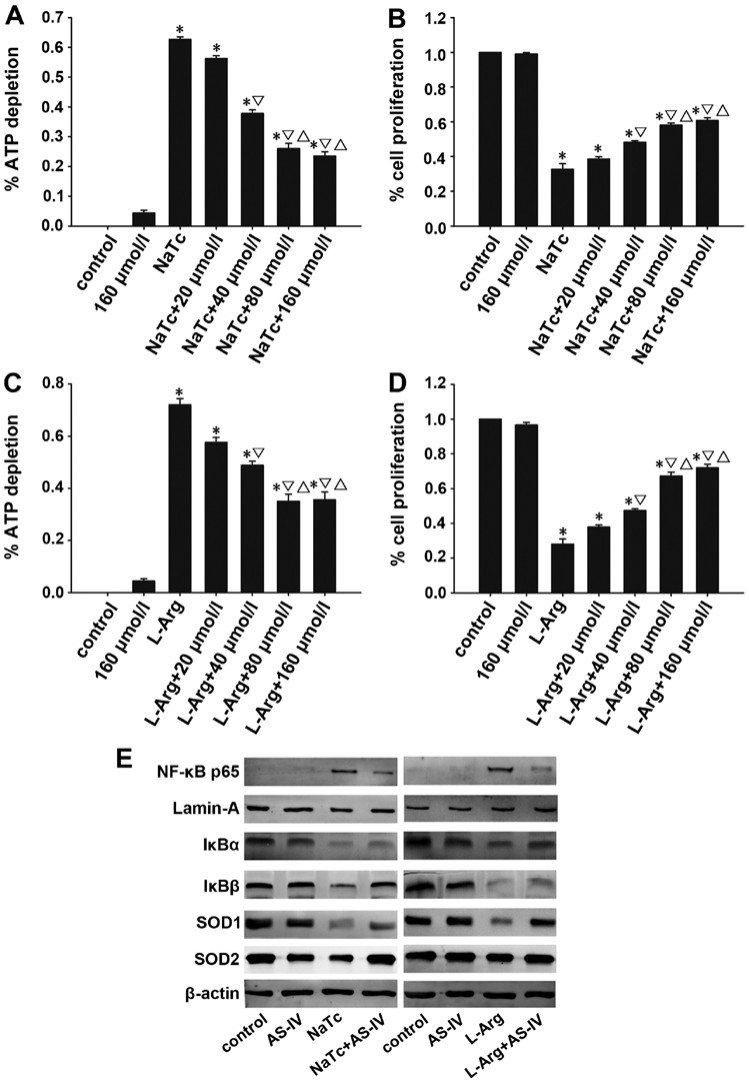
Effects of AS-IV on AP *in vitro*. Necrosis and proliferation of pancreatic acinar cells was detected by the depletion of ATP and CCK-8 assay, respectively. AS-IV suppressed the dose-dependent depletion of ATP in acinar cells treated with NaTc/L-Arg (A and C), and increased the dose-dependent proliferation of acinar cells by CCK-8 (B and D). (E) The expression of NF-κB p65 in the nucleus, and IκBα, IκBβ, SOD1 and SOD2 in the cytoplasm of pancreatic acinar cells was detected by western blot analysis. Data are represented as the means ± SD from 3 independent experiments. ^*^P<0.05 compared with the normal control group at the same time point; ^▽^P<0.05 compared with the NaTc-treated group at the same time point; ^△^P<0.05 compared with the NaTc/L-Arg + 40 *μ*mol/l AS-IV-treated group. AS-IV, astragaloside IV; ATP, adenosine triphosphate; SOD1, manganese superoxide dismutase; SOD2, cuprum/zinc superoxide dismutase; NaTc, sodium taurocholate; L-Arg, L-arginine; AP, acute pancreatitis; NF-κB, nuclear factor-κB; CCK-8, cell counting kit-8.

The depletion of ATP can be used to measure the degree of necrosis of pancreatic acinar cells. The proliferation of pancreatic acinar cells can be detected by CCK-8, a sensitive colorimetric assay. AS-IV alleviated the necrosis of pancreatic acinar cells and improved their viability (Fig. 8A-D). Morever, using western blot analysis, we determined the nuclear protein expression of levels of NF-κB p65 and the cytoplasmic protein expression levels of IκBα, IκBβ, SOD1 and SOD2 (Fig. 8E). As expected, the results were consistent with those of the *in vivo* experiments.

## Discussion

AP is a potentially fatal disease with increasing incidence over the years. The pathogenesis of AP remains to be elucidated despite significant advances over the past 25 years (31). There is an urgent need to develop effective therapeutic options for the treatment of AP. It is generally considered that inflammatory cytokines, leukocytic infiltration, the activation of NF-κB and oxidative stress are key factors in the development of AP (32–34).

Inflammatory mediators play a key role in the pathogenesis of AP and the resultant multiple organ dysfunction syndrome, which is the primary cause of mortality under this condition (5). Pancreatic acinar cells are the primary source of TNF-α during the early phases of AP. In response to TNF-α arising from acinar cells, an intricate sequence of events involving the tissue vasculature and inflammatory cells occur, such as the enhanement of oxidative stress, the assembly of other cytokines and ROS, the abnormal upregulation of adhesion molecules and the promotion of the transmigration of leukocytes into inflamed tissue (11,35,36). All these events are involved in the development of AP and even systemic inflammatory response syndrome (SIRS). Previous studies have suggested that the serum levels of IL-1β correlate with the severity of AP (37,38). A previous study also demonstrated that IL-6 is intimately linked with pancreatic necrosis and other organ dysfunction in experimental pancreatitis (39). The serum levels of IL-6 are significantly higher in SAP compared with mild AP (40,41). Thus, IL-6 is an evaluating indicator of the severity of AP. It has been suggested that the early suppression of these pro-inflammatory cytokines relieves the development of AP and ameliorates the severity of AP (42). The initial injury associated with AP is closely followed by the second stage, namely, the excessive transmigration of leukocytes into inflamed tissue, which plays a crucial role in pancreatic injury and systemic complications. The extent of neutrophil infiltration in the pancreas is quantified by measuring tissue MPO activity (43). It has been confirmed that AS-IV plays an important role in treating inflammatory diseases (18). The anti-inflammatory effects of AS-IV may be mediated through the inhibition of the activition of NF-κB, and activated NF-κB promotes the expression of TNF-α, IL-1β and IL-6 (19,44). In this study, we investigated the effects of AS-IV in 2 well-characterized models of AP induced by NaTc/L-Arg in rats; AP in rats is similar to human AP due to the rapid development of inflammation. Our results demonstrated that AS-IV significantly ameliorated the pancreatic damage in NaTc/L-Arg-induced AP as shown by histological characteristics, MPO activity, and serum amylase and lipase levels. Morever, AS-IV reduced the serum levels of pro-inflammatory cytokines, such as TNF-α, IL-1β and IL-6 and inhibited the mRNA expression levels of TNF-α, IL-1β and IL-6 in the pancreas.

The transcription factor NF-κB is a key factor in the development of AP based on its ability to regulate the expression of inflammatory mediators (32). It has been confirmed that the activation of NF-κB occurs in pancreatic acinar cells in the initial course of AP, and plays a role in the inflammatory response during AP (4,33). NF-κB belongs to the Rel family and exists as a heterodimer or a homodimer formed by polypeptides p50 and p65; RelA/p65 is the crucial transcription factor of the classical pathway of NF-κB (45–47). In general, the inhibitor protein of NF-κB (IκB) keeps NF-κB in the cytoplasm by masking its nuclear localization sequence (48). In response to extracellular stimuli, IκB proteins become hyperphosphorylated, ubiquitinated and degraded in proteosomes (48). Free NF-κB translocates to the nucleus and binds to its specific site-κB sequences, leading to the massive transcription of a variety of genes important for inflammation, including TNF-α, IL-1β and IL-6 (14, 49). In light of these findings, the NF-κB/IκB system in the pancreas presents a novel and exciting potential target for the treatment of AP. It has been found that AS-IV inhibits the activition of NF-κB *in vitro* (19). In this study, we investigated whether AS-IV alleviates the severity of AP by inhibiting the activation of NF-κB. The results from western blot analysis revealed that NF-κB was activated at 12, 24 and 48 h following the induction of AP, particularly at 12 h. However, the administration of AS-IV significantly suppressesed the degradation of IκBα and IκBβ, thereby decreasing the expression of NF-κB p65 in the nucleus during AP. The results from immunohistochemistry further revealed that AS-IV significantly inhibited the staining intensity of nuclear NF-κB p65 in the pancreas.

Oxidative stress has a significant impact on the pathogenesis of AP (34,50). During AP, the excessive generation of ROS and an inefficient intrinsic antioxidative defense system result in the accumulation of ROS and lipid membrane peroxidation (50). As the primary defense of the antioxidant system, SOD is an important antioxidant enzyme, which specifically detoxifies superoxide radicals to hydrogen peroxide. In this study, the results from RT-qPCR revealed that AS-IV increased the mRNA expression levels of SOD1 and SOD2. Morever, western blot analysis also revealed that AS-IV increased the protein expression of SOD1 and SOD2. These results indicate that AS-IV exerts antioxidant effects on the development of AP. However, the antioxidant mechanisms of action of AS-IV require further investigation.

In addition, we investigated the effects of AS-IV in an *in vitro* model of NaTc/L-Arg-induced AP using pancreatic acinar cells. In the *in vitro* model, we found that the high dose of AS-IV reduced pancreatic acinar cell necrosis and improved the viability of the acinar cells. Furthermore, we detected the protein expression of NF-κB p65 in the nucleus, and the expression of IκBα, IκBβ, SOD1 and SOD2 in the cytoplasm of pancreatic acinar cells by western blot analysis. As expected, the results were consistent with those of the *in vivo* experiments.

In conclusion, our data demonstrate that AS-IV attenuates the severity of NaTc/L-Arg-induced experimental AP in rats. Our results revealed that AS-IV exerted anti-inflammatory effects by inhibiting the activation of NF-κB and suppressing the secretion of pro-inflammatory cytokines, which are the main mechanisms of action of AS-IV in AP. As for the antioxidant effects of AS-IV on AP, further investigations are required. In addition, the results of the *in vitro* experiments were consistent with the results obtained in *in vivo* experiments. These findings provide a basis for the further investigation of the therapeutic role of AS-IV in AP.
